# Complete Genome Sequences of Three Lactiplantibacillus plantarum Strains Isolated from Traditional Iranian Raw Milk Motal Cheese

**DOI:** 10.1128/mra.00479-22

**Published:** 2022-12-07

**Authors:** Saeideh Sadat Fatemizadeh, Lukasz Krych, Josue L. Castro-Mejía, Denitsa Vladimirova Stefanova, Witold Kot, Mohammad Bagher Habibi Najafi, Dennis Sandris Nielsen

**Affiliations:** a Department of Food Science and Technology, Faculty of Agriculture, Ferdowsi University of Mashhad, Mashhad, Iran; b Department of Food Science, Faculty of Science, University of Copenhagen, Copenhagen, Denmark; c Department of Plant and Environmental Sciences, Faculty of Science, University of Copenhagen, Copenhagen, Denmark; University of Maryland School of Medicine

## Abstract

The complete genome sequences, as determined by a combination of short- and long-read sequencing, of three Lactiplantibacillus plantarum strains (M8, M17, and M19) that were isolated from Iranian motal cheese are reported. The genome sizes were estimated to be 3.3, 3.3, and 3.5 Mbp, respectively, with GC contents of approximately 44.5%.

## ANNOUNCEMENT

Whole-genome sequencing was applied to three strains of Lactiplantibacillus plantarum that had been previously isolated from motal cheese (1, 2). Pure isolates were restreaked on MRS agar (Merck, Germany) for 48 h at 37°C, propagated on MRS agar for 24 h at 30°C, and harvested by centrifugation (5 min) at 12,000 × *g* at 5°C. DNA was extracted using the Bead-Beat Micro AX Gravity kit (A&A Biotechnology, Poland).

For short-read sequencing, the Nextera XT library preparation kit (Illumina) was used to generate libraries from 1 ng DNA. Libraries were cleaned (AMPure XP beads; Beckman Coulter) and sequenced (NextSeq 550 system, with 2 × 150-bp paired-end reads). Reads were trimmed using Trimmomatic v0.39 (3) and quality checked using FastQC v0.11.7 and MultiQC v1.2.

Oxford Nanopore Technologies (ONT) libraries were prepared with a rapid barcoding sequencing kit (SQK-RBK004; ONT) and sequenced on an ONT GridION system. NanoFilt v2.2.0 and NanoStat v0.8.1 were used for trimming and quality control, respectively (4).

An average of 288 Mbp (standard deviation [SD], 30 Mbp) of short-read sequences per isolate and an average of 451 Mbp (SD, 68 Mbp) of long-read sequences per isolate were hybrid assembled *de novo* into circular chromosomes using Unicycler v0.4.8, which provides oriented assemblies, decreasing the risk of splitting genes (at the start and end of the sequence) by searching for *dnaA* and *repA* (initiation/replication genes) and rotating/flipping the sequence so that it begins with that gene encoded on the forward strand (5). Annotation was performed using RAST v3.6.12 (6) under the taxonomic identification number (Taxid) of L. plantarum (Taxid 1590) and genetic codes of archaea and bacteria. Quality and completeness were evaluated using QUAST v4.6.0 (7) and BUSCO v4 (8) (Odb10 data set), respectively. The 16S rRNA genes were identified (TrueBac ID; ChunLab Inc., South Korea) and aligned using NCBI BLAST for 16S rRNA gene sequences (9). Species identification was based on average nucleotide identity (ANI) comparisons between the isolates and their type strain (L. plantarum strain DSM 20174 (GenBank accession number GCA_014131735.1)) using TrueBac ID. Table 1 contains ANI values and species identification results (10).

**TABLE 1:** *De novo* genome assembly data for chromosomes and plasmids of completely sequenced Lactiplantibacillus plantarum strains from motal cheese

Strain	Illumina sequencing results			ONT sequencing results					Genome size (bp)	No. of contigs	GenBank accession no. (size [bp])	No. of coding sequences	*N*_50_ (bp)	GC content (%)	GenBank assembly accession no.	ANI (%)^*a*^
	No. of reads	Avg read length (bp)	SRA accession no.	No. of reads	Avg read length (bp)	*N*_50_ (bp)	Coverage depth (×)	SRA accession no.								
M8	1,565,877	151	SRX9855952	62,684	3,553.5	3,569	68.96	SRX14468001	3,294,048	2	Chromosome, CP094381.1 (3,244,346); plasmid, CP094382.1 (49,702)	3,176	3,244,346	44.49	GCA_018588665.2	99.22
M17	1,759,165	151	SRX9855953	56,130	3,472.3	3,462	60.34	SRX14468002	3,294,393	2	Chromosome, CP094383.1 (3,244,691); plasmid, CP094384.1 (49,702)	3,178	3,244,691	44.50	GCA_018588615.2	99.23
M19	2,027,154	151	SRX9855954	74,091	3,563.2	3,626	81.73	SRX14468003	3,510,837	11	Chromosome, CP094385.1 (3,153,037); plasmid 1, CP094386.1 (88,918); plasmid 2, CP094387.1 (54,570); plasmid 3, CP094388.1 (51,943); plasmid 4, CP094389.1 (40,748); plasmid 5, CP094390.1 (38,996); plasmid 6, CP094391.1 (29,277); plasmid 7, CP094392.1 (17,237); plasmid 8, CP094393.1 (14,169); plasmid 9, CP094394.1 (13,244); plasmid 10, CP094395.1 (8,698)	3,509	3,153,037	44.35	GCA_018588605.2	98.90

a ANI (%) represents comparison with the type strain.

Data comparisons using CARD (11), ARG-ANNOT (12), and ResFinder (https://cge.food.dtu.dk/services/ResFinder) revealed no antibiotic resistance genes or virulence factors (13). The PlasmidFinder online tool v2 detected 1 plasmid in strains M8 and M17 and 10 plasmids in M19. Unless specified, default parameters were used for all analyses.

### Data availability.

Complete genome assemblies are available under BioProject accession number PRJNA692080.
